# A Compact Button Antenna with Dual-Band and Dual-Polarization for Wearable Body Area Networks

**DOI:** 10.3390/mi17010028

**Published:** 2025-12-26

**Authors:** Xue-Ping Li, Zhen-Yong Dong, Xue-Qing Yang, Meng-Bing Yang, Xiao-Ya Li, Xi-Qiao Wu, Wei Li

**Affiliations:** 1 School of Optoelectronic Engineering, Henan Normal University, Xinxiang 453600, China; lixueping@htu.edu.cn (X.-P.L.); 13938479502@163.com (Z.-Y.D.); 2322324016@stu.htu.edu.cn (X.-Q.Y.); 15886778141@163.com (M.-B.Y.); 17527186689@163.com (X.-Y.L.); 2 Henan Key Laboratory of Optoelectronic Sensing Integrated Application, Henan Normal University, Xinxiang 453600, China

**Keywords:** button antennas, circularly polarized antenna, wearable antenna, wireless body-area networks (WBANs)

## Abstract

This paper presents a compact, dual-band, dual-polarization button antenna for Wireless Body Area Networks (WBANs) that operates in the 2.45 GHz and 5.8 GHz Industrial, Scientific, and Medical (ISM) bands. The antenna is engineered in the lower band from 2.33 to 2.8 GHz (18.3% fractional bandwidth) as a linearly polarized, top-loaded monopole, which provides an omnidirectional radiation pattern for on-body communication. In contrast, it functions as a cross-dipole in the higher band, achieving a fractional bandwidth of 66.4% (4.8–9.57 GHz) and a 3 dB axial ratio (AR) bandwidth of 57.4%, producing a broadside radiation with circular polarization for off-body communications. Prototype measurements in both free-space and on-body settings confirm the antenna’s robust performance, successfully validating its dual-band operation, dual-polarization characteristics. Furthermore, Specific Absorption Rate (SAR) simulations conducted on a human model demonstrate that the values are significantly below the established safety limits, confirming the antenna’s suitability for practical wearable applications.

## 1. Introduction

The proliferation of wireless networks and the ongoing miniaturization of electronic devices have catalyzed the development of wireless body area networks (WBANs), leading to diverse wearable applications in healthcare, sports, and the military [1,2,3,4,5]. As the cornerstone of these systems, wearable antennas must evolve to meet new requirements and design challenges posed by complex on-body environments and diverse application scenarios. For operate effectively in complex on-body environments, they are often designed to be flexible. For instance, many studies employ textile-based planar structures that can be seamlessly integrated into clothing [6,7,8,9]. Alternatively, other designs have integrated antennas into common accessories, including eyeglass frames [10], bag zippers [11], wrist straps [12], and berets [13]. This integration technique not only enhances compatibility but also effectively fulfills the data transmission demands of WBANs.

Among various wearable antennas, the button antenna presents significant advantages in the wearable landscape, primarily due to their rigid, stable form factor and a profile that facilitates clothing integration while countering performance degradation from movement. These benefits have spurred considerable research interest. Initial designs, such as the dual-frequency dipole array in [14], even incorporated practical fastening functions. A central challenge in wearables is mitigating polarization mismatch and multipath loss which is arising from postural changes or antenna deformation. To address this, circular polarization (CP) has been effectively employed. As demonstrated by a novel open-complementary CP antenna for the 5.47–5.725 GHz U-NII band [15] and an omnidirectional CP design using a magnetic dipole combined with a flexible patch on textile for 5 GHz [16]. Furthermore, driven by the need for miniaturization and multi-functionality in Internet of Things (IoT), research has progressed towards more integrated solutions. A capacitively fed dual-band button antenna with a compact volume and high radiation efficiency is proposed in [17]. Ref. [18] propose a dual-band dual-mode button antenna utilizing a helical inverted-F structure, which exhibits omnidirectional radiation at 2.4 GHz and directional radiation at 5.8 GHz. A dual-band dual-polarization button antenna is reported in [19], and it employs metal rivets to achieve omnidirectional vertically polarized radiation at 2.45 GHz for on-body communication and directional circularly polarized radiation at 5.8 GHz for off-body communication. Additionally, the design for WBANs in [20] adopts the use of parasitic patches to enhance bandwidth in both the lower and upper bands. Furthermore, these diverse design strategies collectively propel the continued advancement of WBAN antennas in terms of performance optimization and application scenario adaptability.

In this work, a dual-band dual-polarization button antenna is designed to fulfill the distinct requirements of both on- and off-body communication. The antenna’s high-frequency mode provides directional broadside radiation with a 57.4% 3 dB axial ratio (AR) bandwidth, making it highly effective for off-body communication. For the lower band, impedance matching at 2.45 GHz is accomplished via quarter-wavelength phase-shifting loops of varying widths, enabling the structure to function as a capacitively top-loaded monopole that radiates an omnidirectional, linearly polarized (LP) pattern for on-body communication. Experimental characterization of a prototype in both free space and on-body conditions validates the intended dual-band operation, dual-polarization characteristics, and overall suitability for practical wearable applications.

## 2. Antenna Geometry

**Figure 1:**
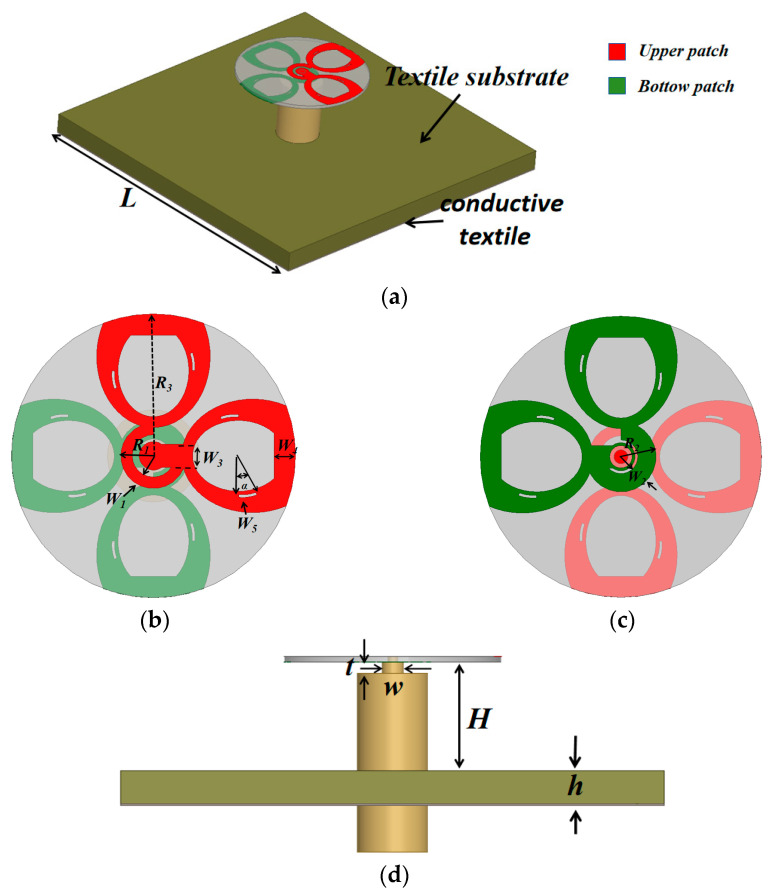
Antenna structure diagram; (**a**) Top view; (**b**) Front view and; (**c**) Bottom view (**d**) Side view.

Figure 1 depicts the configuration of the proposed single-port button antenna. The antenna design comprises a radiating button head fabricated on a 0.508 mm-thick F_4_BM220 substrate (εᵣ = 2.2, tan δ = 0.0009), a rigid coaxial feed probe that also serves as mechanical support, and a metallized textile ground plane. The radiating element is fed through an open quarter-wavelength phase-shifting line. Specifically, the upper monopole patch is connected to the inner conductor of the coaxial cable, while an identical but 180°-rotated structure on the bottom layer of the substrate is connected to the outer conductor. To prevent a short circuit, the outer conductor is tapered at the feed point (situated 1 mm below the substrate), as its original radius exceeds that of the feeder delay line (R_1_), a feature detailed in Figure 1d. The entire button head assembly is positioned over a 3 mm-thick textile substrate (εᵣ = 1.4, tan δ = 0.044) with a central 10 mm air gap, facilitating integration into garments. Moreover, the structure is backed by a 50 mm × 50 mm ground plane made of SHIELDIT™ SUPER conductive textile (thickness: 0.17 mm, conductivity: 1.18 × 10^5^ S/m, Dahuakangqiao Ltd. (Shiyan, China)), a material widely recognized for shielding and commonly used as a conductor in textile antennas.

## 3. Design Strategy and Working Principle of Antenna

The design of a dual-band, dual-polarized wearable button antenna must overcome two primary challenges. The first is the development of a compact, dual-band radiating structure capable of seamless integration with a larger textile ground plane on a garment. A second, more fundamental challenge concerns polarization efficiency. While LP antennas are commonly used due to their simplicity, their performance is highly vulnerable to signal degradation from unpredictable user movements and posture changes. CP antennas, in contrast, are inherently more robust to such orientation misalignments, as the rotational nature of their waves minimizes polarization loss and signal fading. However, achieving CP radiation in a compact button antenna is significantly more complex than implementing LP operation. Given this trade-off, our design strategy prioritizes the realization of a stable CP directional radiation pattern in the high band before addressing the LP omnidirectional requirement for the low band.

The proposed button antenna is built upon a conventional crossed-dipole architecture. A critical element for generating CP is the integration of open quarter-wavelength phase-shifting lines, which connect the radiating patches on the top and bottom substrate surfaces. When the electrical length of this line equals a quarter-wavelength at the target frequency, it can introduce the essential 90° phase shift between two orthogonal dipole modes. The phase difference is primarily governed by the geometric parameters (R_1_, W_1_) of the phase-shifting line, with its electrical length approximated as Lphase shift = 2π × (R_1_ − W_1_/2) × 3/4, where R_1_ and W_1_ are geometric parameters defined in Table 1. While conventional dipole designs with narrow, rectangular arms suffer from limited axial ratio bandwidth (ARBW), this work employs modified elliptical dipole arms, which is known to achieve enhanced ARBW compared to traditional tapered or flared designs.

**Figure 2:**
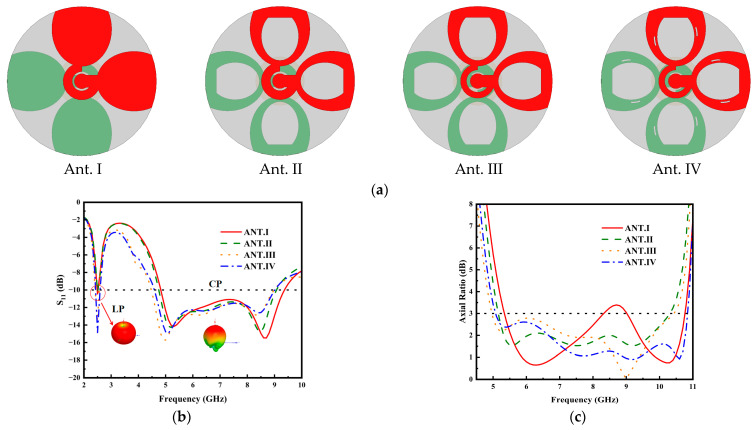
(**a**) Antenna evolution process; (**b**) Corresponding S-parameters; (**c**) Higher-band axial ratios.

The evolutionary stages of the antenna design are illustrated in Figure 2. The initial design (Ant. I) offers a wide fractional bandwidth in the upper band but suffers from poor AR performance. To address this, an incomplete elliptical slot is incorporated into the radiating patch, resulting in Ant. II. This slot suppresses reverse currents, thereby significantly improving the AR performance without compromising the upper-band impedance matching. Ant. II achieves wide impedance bandwidth (|S_11_| < −10 dB) and AR (AR < 3 dB) bandwidth is 4.70–9.20 GHz and 5.10–11.05 GHz but exhibits poor matching at 2.45 GHz. Subsequently, the width of the top phase-shifting ring is reduced to create Antenna III (Ant. III). This asymmetry in ring widths reduces the reactance near 2.45 GHz, successfully yielding a well-matched lower band (2.41–2.57 GHz) while also enhancing the upper-band matching. In the final design (Ant. IV), curved slots are introduced at various positions of the radiating patch. These slots create additional surface current paths that compensate for the phase delay of the fixed-length phase-shifting line, resulting in a significant enhancement of polarization purity within the operational band.

**Figure 3:**
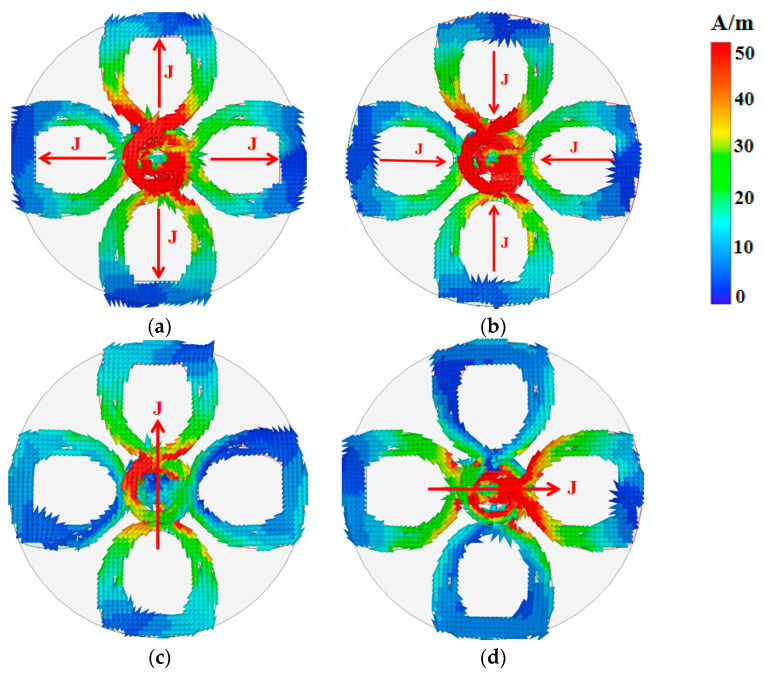
Instantaneous multilayer current distribution on the button head 2.45 GHz (**a**) 1/4 T; (**b**) 3/4 T; 5.8 GHz (**c**) 1/4 T; (**d**) 3/4 T. The red arrows are added on the most active dipole arms for visual guidance.

Figure 3 illustrates the surface current distributions on the button antenna at 2.45 GHz and 5.8 GHz. At lower frequencies, the coaxial cable and the button head collectively function as a conventional monopole antenna, loaded with a circular plate situated above a textile ground plane. As shown in Figure 3a,b, the current radiates uniformly inward or outward on the elliptical patch and moves back and forth within one cycle. This current distribution pattern determines the antenna’s radiation characteristics, indicating the generation of an omnidirectional, LP radiation pattern. For the upper band at 5.8 GHz (Figure 3c,d), the operation shifts to a crossed-dipole mode. The current magnitude peaks at the centers of the dipoles (i.e., the feed points) and diminishes toward their extremities. A distinct counterclockwise rotation of the current vector along the axis (marked by red arrows) confirms the generation of right-hand circularly polarized (RHCP) radiation. This is quantitatively demonstrated by the radiation pattern, presented in Figure 2b. Consequently, the antenna produces a directional circularly polarized beam along the +*Z*-axis, making it ideal for off-body communication links.

## 4. Results and Discussion

**Figure 4:**
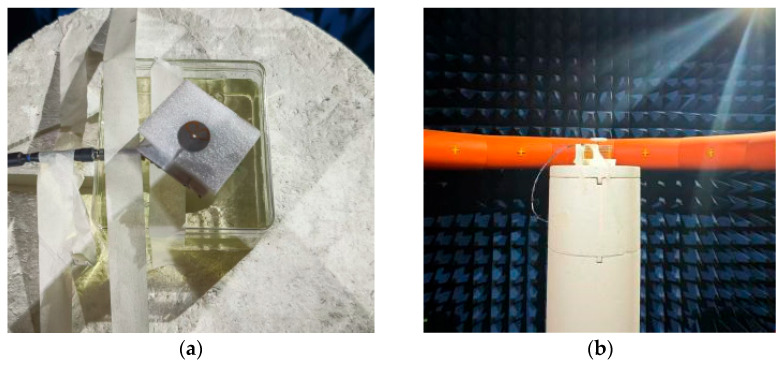
The actually fabricated antenna: (**a**) Top View; (**b**) Measurement setup in an echoic chamber.

The operational environment of a wearable antenna differs significantly from free space, as the high permittivity and loss tangent of the human body can detune the resonant frequency and reduce radiation efficiency. To validate the proposed design’s robustness, prototypes were fabricated as Figure 4 and their performance was characterized under both free space and mounted on the human body. The antenna is fed by a coaxial cable via an SMA connector. For practical wearable integration, the antenna can be directly connected to a compact transceiver module located behind its structure.

**Figure 5:**
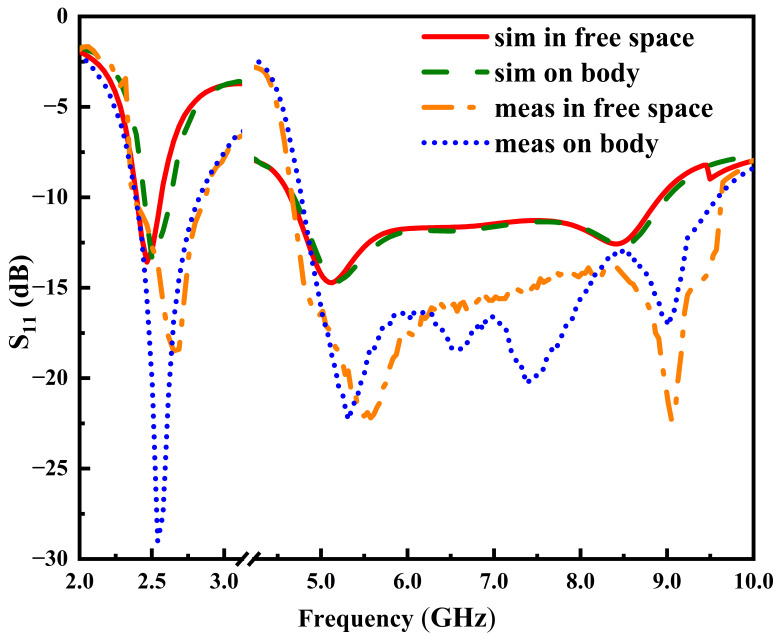
Measured and simulated reflection coefficients of the button antenna in free space and on body.

Figure 5 presents the simulated and measured reflection coefficients S_11_ of the proposed button antenna under two operating conditions, demonstrating excellent agreement with the intended dual-band operation in the 2.45 GHz and 5.8 GHz WBAN bands. The antenna exhibits relative stability in the presence of the human body, with minimal performance degradation. This robustness is credited to the conductive ground plane, which effectively shields the antenna from the body by suppressing backward radiation. However, a slight improvement in impedance matching is observed in the upper band, potentially due to energy absorption by the human tissue. The measured −10 dB impedance bandwidths are 470 MHz (2.33–2.8 GHz, 18.3% fractional bandwidth) for the lower band and 4.77 GHz (4.8–9.57 GHz, 66.4% fractional bandwidth) for the upper band, which are broadly consistent with the simulated results. The discrepancies between simulation and measurement are likely attributable to manufacturing tolerances, slight excess solder, and variations in the coaxial cable placement during testing.

**Figure 6:**
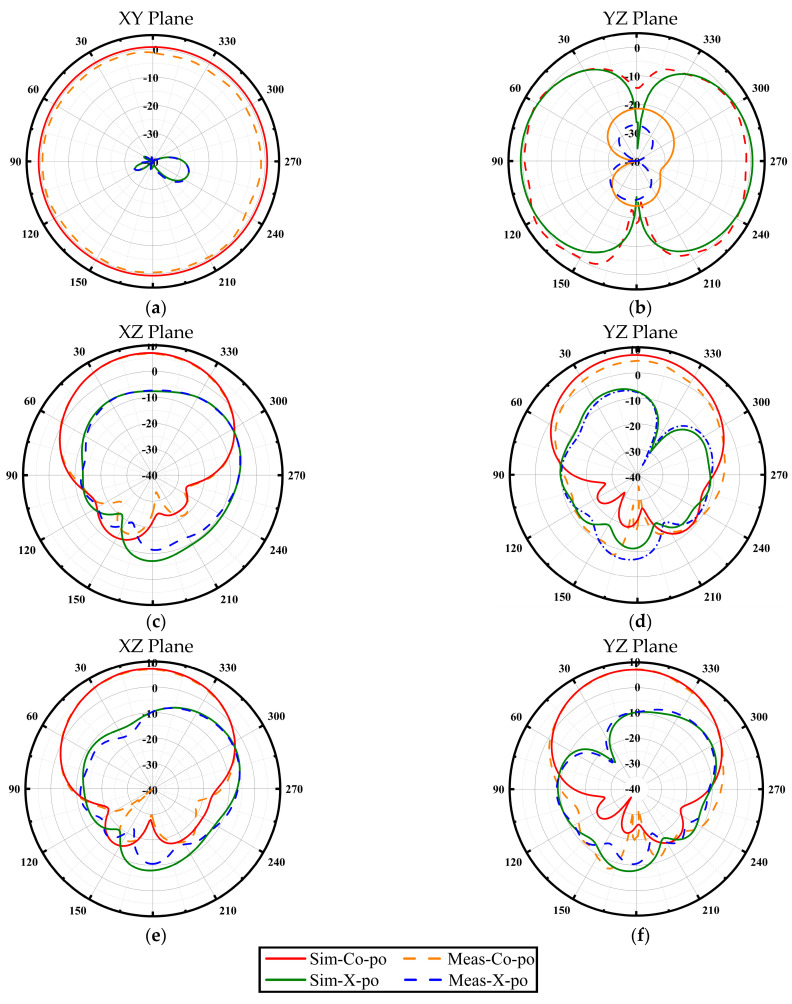
Measured and simulated normalized radiation patterns of the button antenna; (**a**,**b**) 2.45 GHz; (**c**,**d**) 5.2 GHz; (**e**,**f**) 5.8 GHz.

Figure 6 shows the simulated and measured 2D radiation patterns of the button antenna at 2.45 GHz, 5.2 GHz and 5.8 GHz. A strong agreement between simulation and measurement is observed. At 2.45 GHz, the antenna exhibits a classic omnidirectional pattern with vertical polarization, which is ideal for establishing on-body communication links around the user. In the upper band (5.2 GHz and 5.8 GHz), the pattern shifts to directional, broadside radiation with significantly suppressed backside levels. This characteristic is crucial for off-body communication, as it efficiently directs energy away from the user, thereby minimizing power absorption into the body. The presence of the large conductive ground plane is key to this performance, effectively isolating the antenna from the human body and ensuring pattern stability.

**Figure 7:**
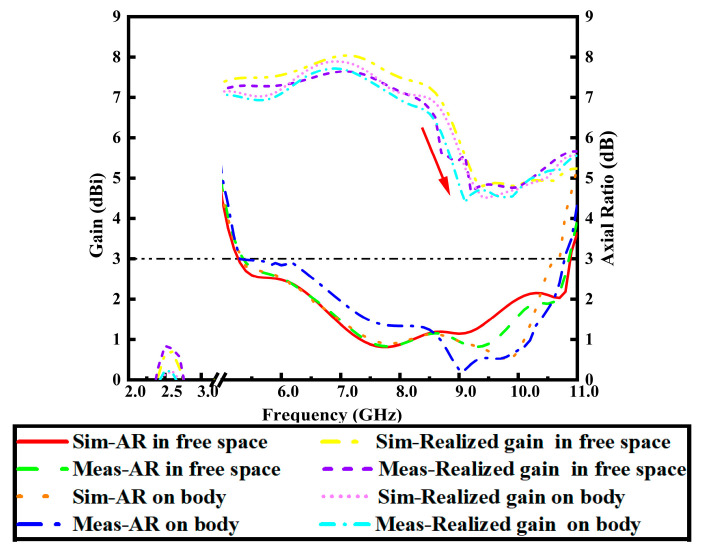
Realized gain and AR at boresight (θ = 0°) in the two operation bands.

Figure 7 presents the simulated and measured AR and realized gain of the proposed antenna in both free space and on-body scenarios. It can be observed that the gain and AR performance across the entire AR bandwidth show minimal variation under the two tested environments. A key observation is the rapid decline in gain within the upper CP band (highlighted by red arrows), particularly around 9.0 GHz. The reason lies in the design of the coaxially fed antenna, whose height above the textile ground plane is set to approximately a quarter-wavelength in free space at 5.8 GHz. This spacing allows the ground plane to effectively reflect backward cross-polarized radiation. As the frequency increases, the required quarter-wavelength shortens correspondingly. Beyond approximately 8.5 GHz, this fixed height becomes insufficient for sustaining effective boresight reflection, resulting in a sharp gain drop in the upper frequency band. In the lower band, the on-body measured gain is only 0.3 dBi, a reduction attributable to the proximity of the human body. Measurement results show that the 3 dB AR bandwidth within the operational band ranges from 5.2 GHz to 9.57 GHz in free space, and from 5.3 GHz to 9.57 GHz in on-body conditions. Minor discrepancies between simulation and measurement are consistent with the idealized nature of the simulation model and the practical tolerances of manual fabrication.

**Figure 8:**
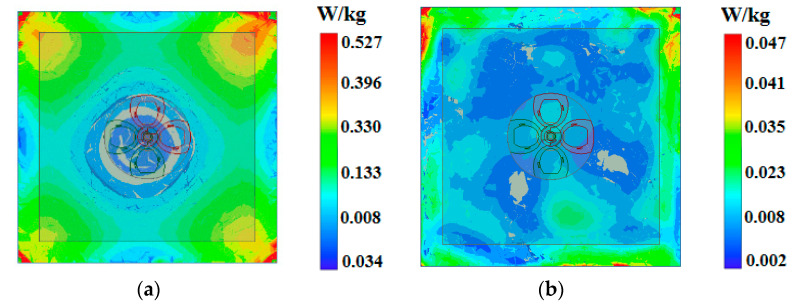
Distribution of SAR on the simulated human tissue model. (**a**) 2.45 GHz; (**b**) 5.8 GHz.

The Specific Absorption Rate (SAR) values induced by the proposed antenna is evaluated through simulation using a 60 × 60 × 28 mm^3^ human tissue model, following the IEEE/IEC 62704-4:2020 [21]. The antenna is positioned 10 mm from a human tissue model to simulate the realistic scenario of a wearable button device on the body. As illustrated in Figure 8, the SAR distributions are computed for input powers of 0.5 W at the operating frequencies of 2.45 GHz and 5.8 GHz. The SAR values, calculated over 1 g of tissue, are 0.527 W/kg at 2.45 GHz and 0.047 W/kg at 5.8 GHz. Both values are well below the stringent safety limit of 1.6 W/kg, confirming the antenna’s compliance with international regulatory standards and its suitability for safe wearable applications.

A comprehensive performance comparison between the proposed antenna and state-of-the-art button antennas is summarized in Table 2, covering key metrics such as electrical size, bandwidth, polarization diversity, radiation patterns, and realized gain. While the proposed design has a slightly larger profile than some references, its dimensions remain well within the standard for clothing fasteners. More importantly, it demonstrates highly competitive performance. It achieves a broader impedance bandwidth than the multi-pattern designs in [17,18,20] and features a superior AR bandwidth of 57.4% in the high band. When compared to the capacitively loaded cross-dipole in [19], our design offers a significantly wider AR bandwidth, albeit with a moderately lower gain. Both designs share a similar coaxial-fed compact form factor, but our implementation utilizes the cheaper F4BM220 substrate, which is much cheaper than that of [19].

## 5. Conclusions

This paper has introduced a wearable, dual-band, dual-polarized button antenna for WBANs, which is simulated, fabricated, and experimentally validated. The proposed design features a compact form factor and demonstrates a favorable impedance bandwidth alongside stable far-field radiation patterns. The design provides omnidirectional linear polarization at 2.45 GHz for on-body networks and directional circular polarization with a 57.4% AR bandwidth in the 5 GHz band for off-body links. Furthermore, the integration of a conductive fabric ground plane ensures both stable performance in wearable scenarios and reduced electromagnetic exposure to the user. These combined attributes establish the antenna as a highly promising and integrated solution for next-generation WBAN applications.

## Figures and Tables

**Table 1:** Antenna parameters.

Parameters	Value (mm)	Parameters	Value (mm)
R_1_	1.5	W_5_	3
R_2_	1.2	w	0.8
R_3_	10	t	1
W_1_	0.8	H	10
W_2_	1.3	h	3
W_3_	1.6	L	50
W_4_	1.5	α (deg)	25

**Table 2:** Comparison table of typical button antennas.

Ref.	Radius/ Height	−10 dB |S11| BW(GHz)	Polarization /3 dB-AR BW(GHz)	Radiation Pattern	Gain (dBi)
[17]	0.16λ_0_/0.15λ_0_	2.38–2.52 (6%) 4.92–6.9 (36%)	LP	O & D	0.3/5.5
[18]	0.15λ_0_/0.18λ_0_	2.40–2.44 (2%) 5.80–6.0 (3%)	LP	O & D	1.0/6.4
[19]	0.20λ_0_/0.22λ_0_	2.38–2.53 (7%) 4.84–8.92 (52%)	LP & CP 5.72–7.85 (31.4%)	O & D	2.2/8.6
[20]	0.21λ_0_/0.24λ_0_	2.3–2.49 (7.9%) 5.15–7.71 (39.8%)	LP	O & D	2.1/5.7
This work	0.22λ_0_/0.23λ_0_	2.33–2.8 (18.3%) 4.8–9.57 (66.4%)	LP & CP 5.3–9.57 (57.4%)	O & D	0.3/7.7

## Data Availability

The original contributions presented in this study are included in the article. Further inquiries can be directed to the corresponding author.
